# Supplementary material for: Interaction network of human early embryonic transcription factors
Source: EMBO Rep. 2024 Jan 31;25(3):1589–622. doi: 10.1038/s44319-024-00074-0 (PMC10933267; doi:10.1038/s44319-024-00074-0)
Supplement: Supplementary file 1 — Appendix [file 44319_2024_74_MOESM1_ESM.pdf]

Interaction network of human early embryonic transcription factors

Lisa Gawriyski 1,2,3, Zenglai Tan* 4, Xiaonan Liu* 1, Iftekhar Chowdhury* 1, Dicle Malaymar-Pinar* 1,5 , Qin Zhang 6, Jere Weltner 2,3 , Eeva-Mari Jouhilahti 2,3 , Gong-Hong Wei 4,6 , Juha Kere 2,3,7 , and Markku Varjosalo 1 §

Appendix

Appendix Figure S1--2
Appendix Figure S2--3
Appendix Figure S3--4
Appendix Figure S4--5
Appendix Figure S5--6
Appendix Figure S6--7
Appendix Figure S7--8
Appendix Figure legends---9

Appendix Figure S1

A

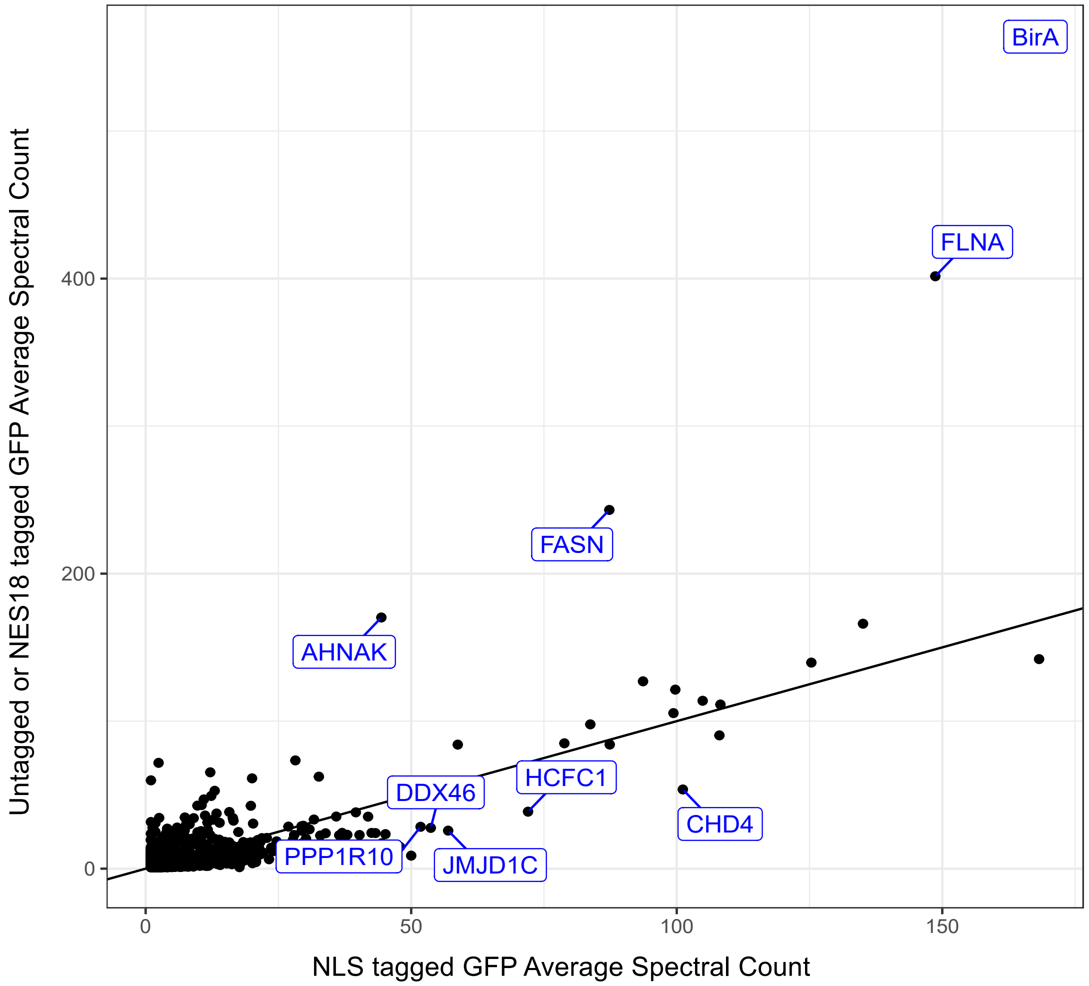

B

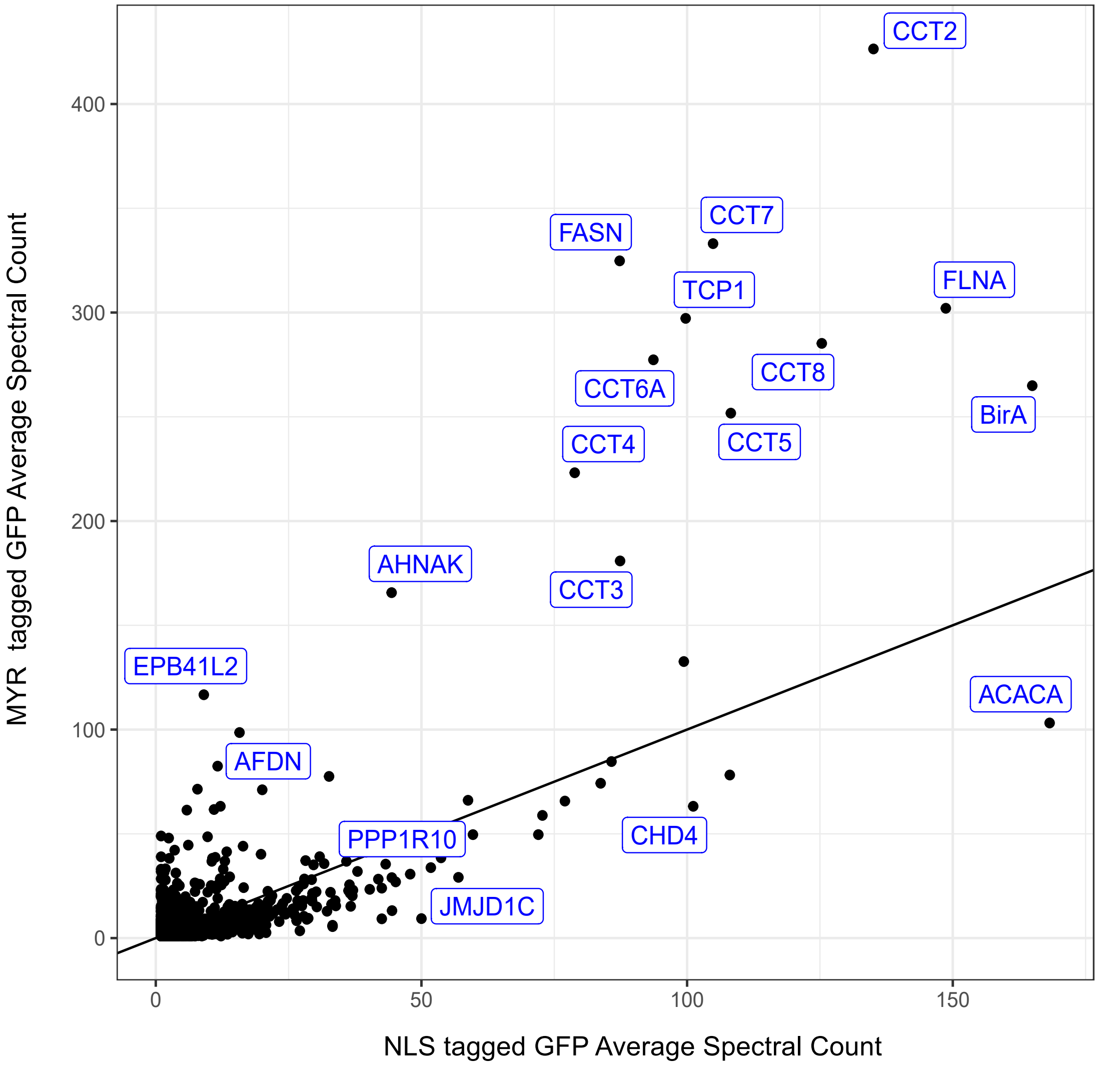

Appendix Figure S2

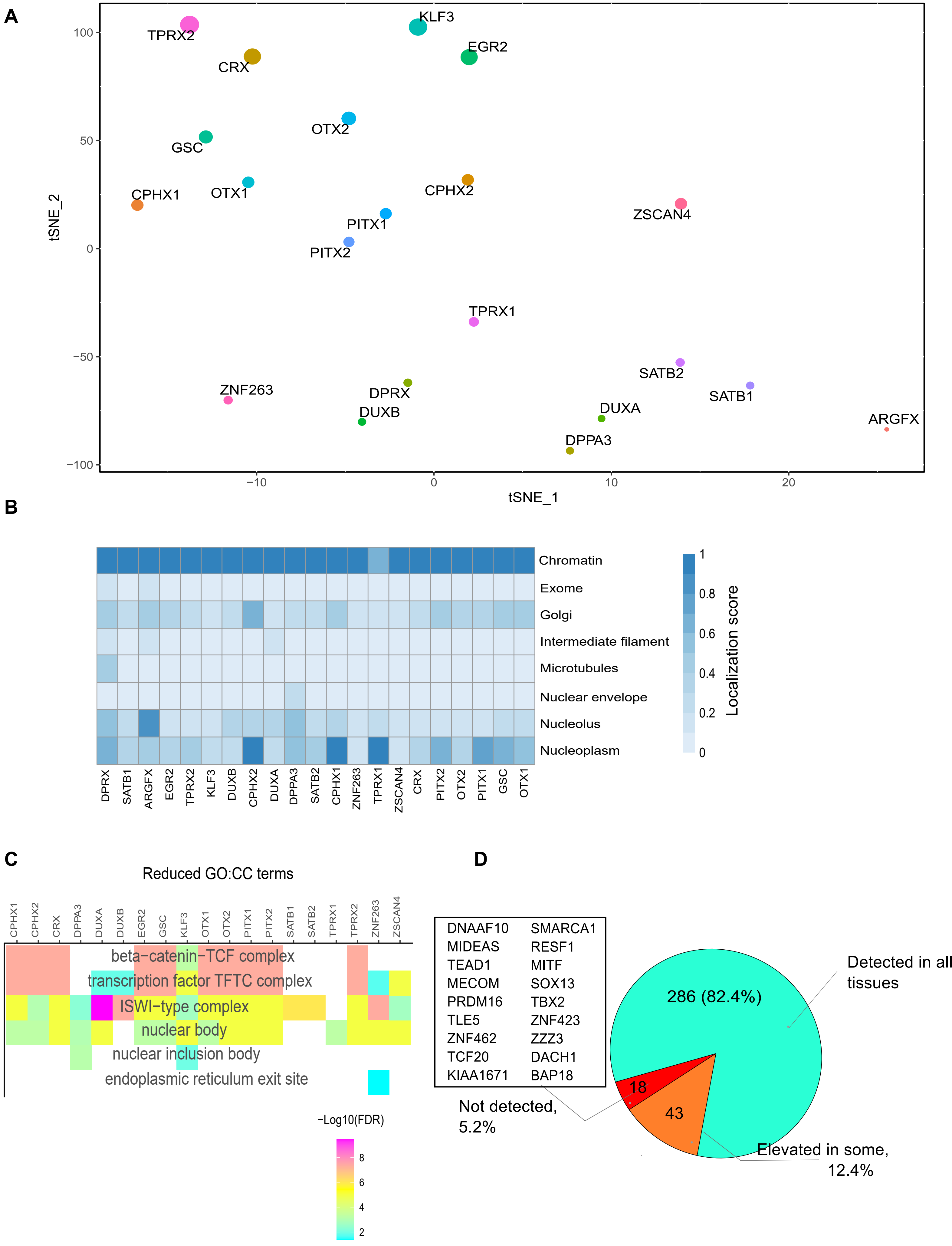

Appendix Figure S3

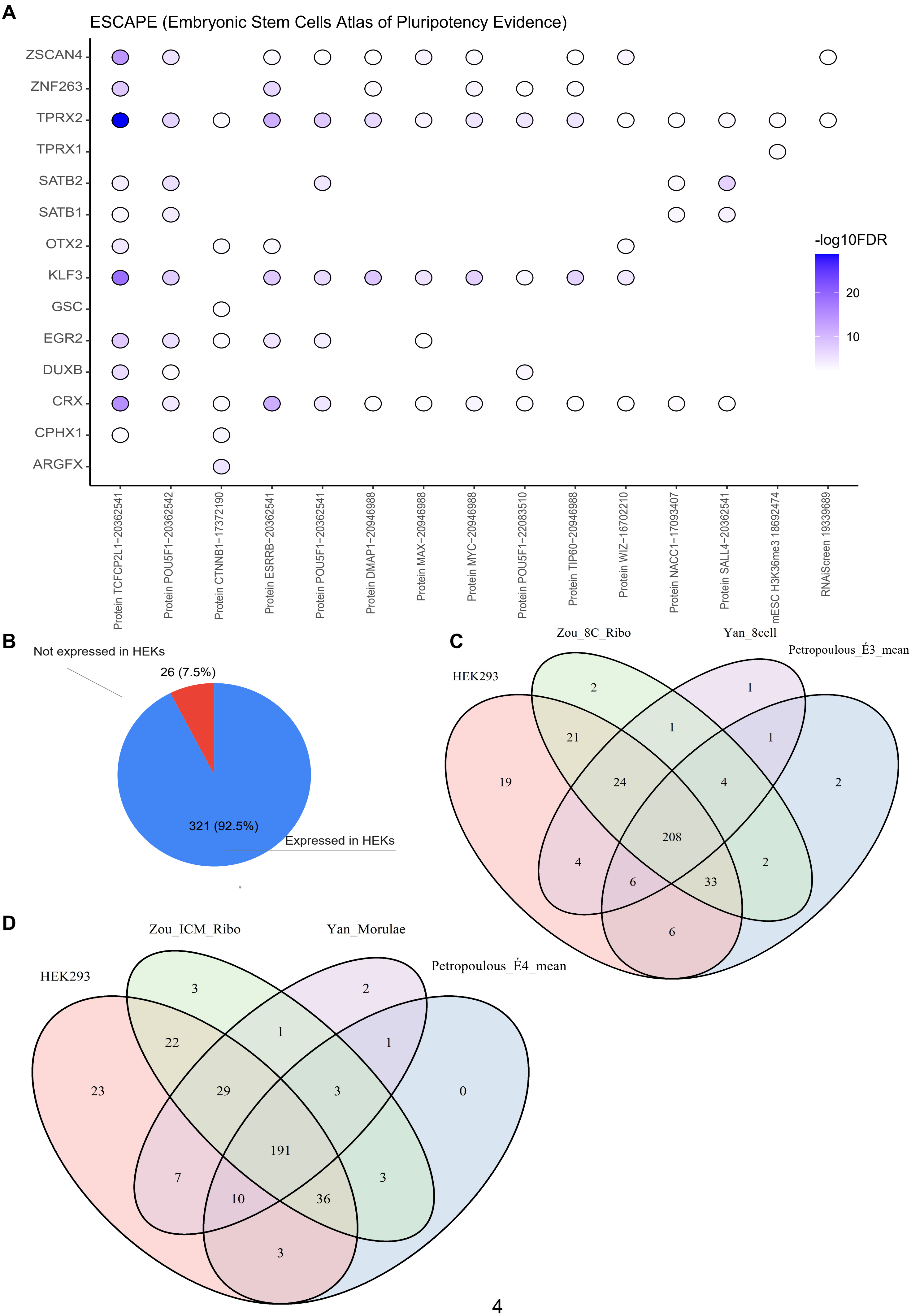

Appendix Figure S4

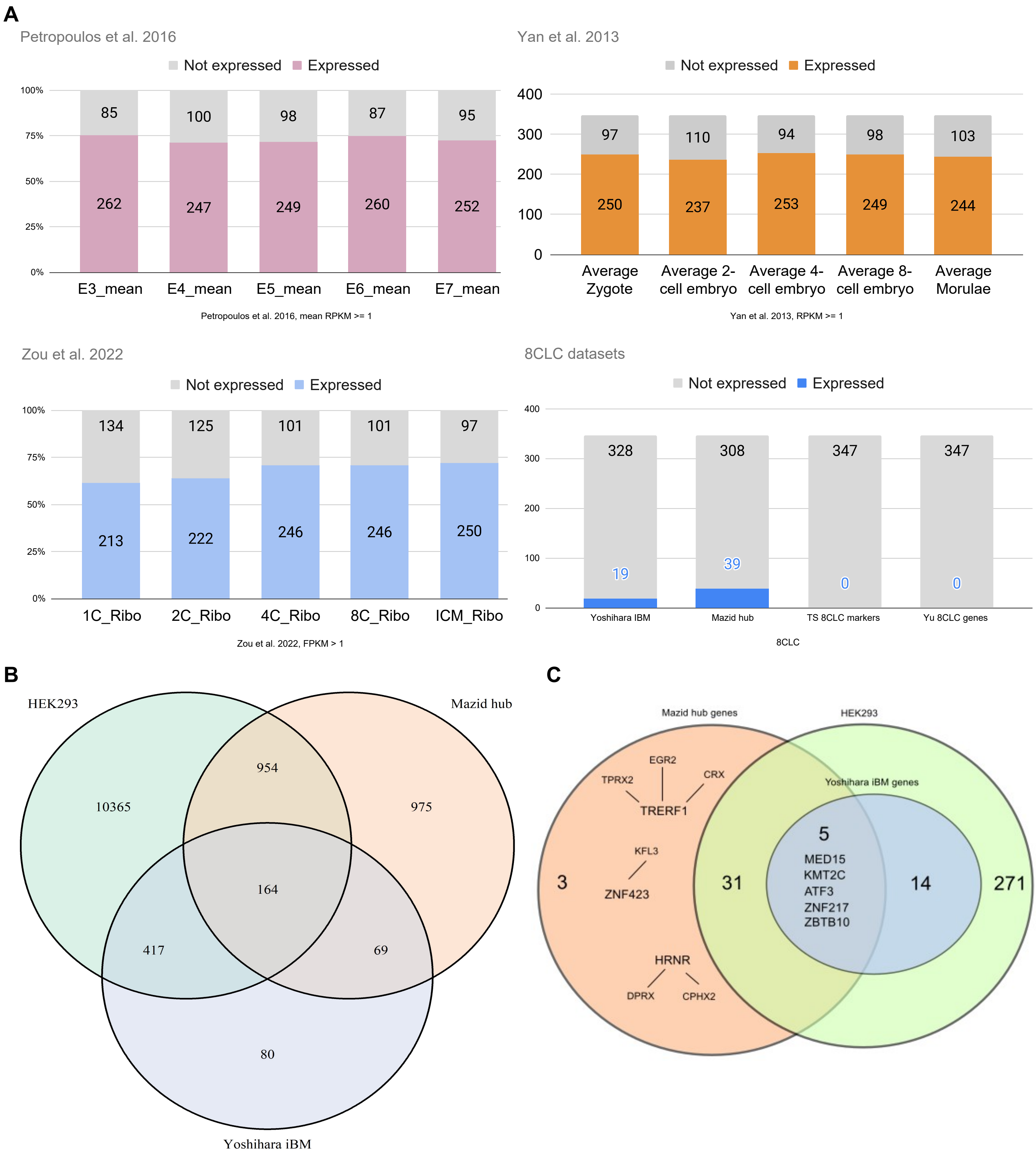

Appendix Figure S5

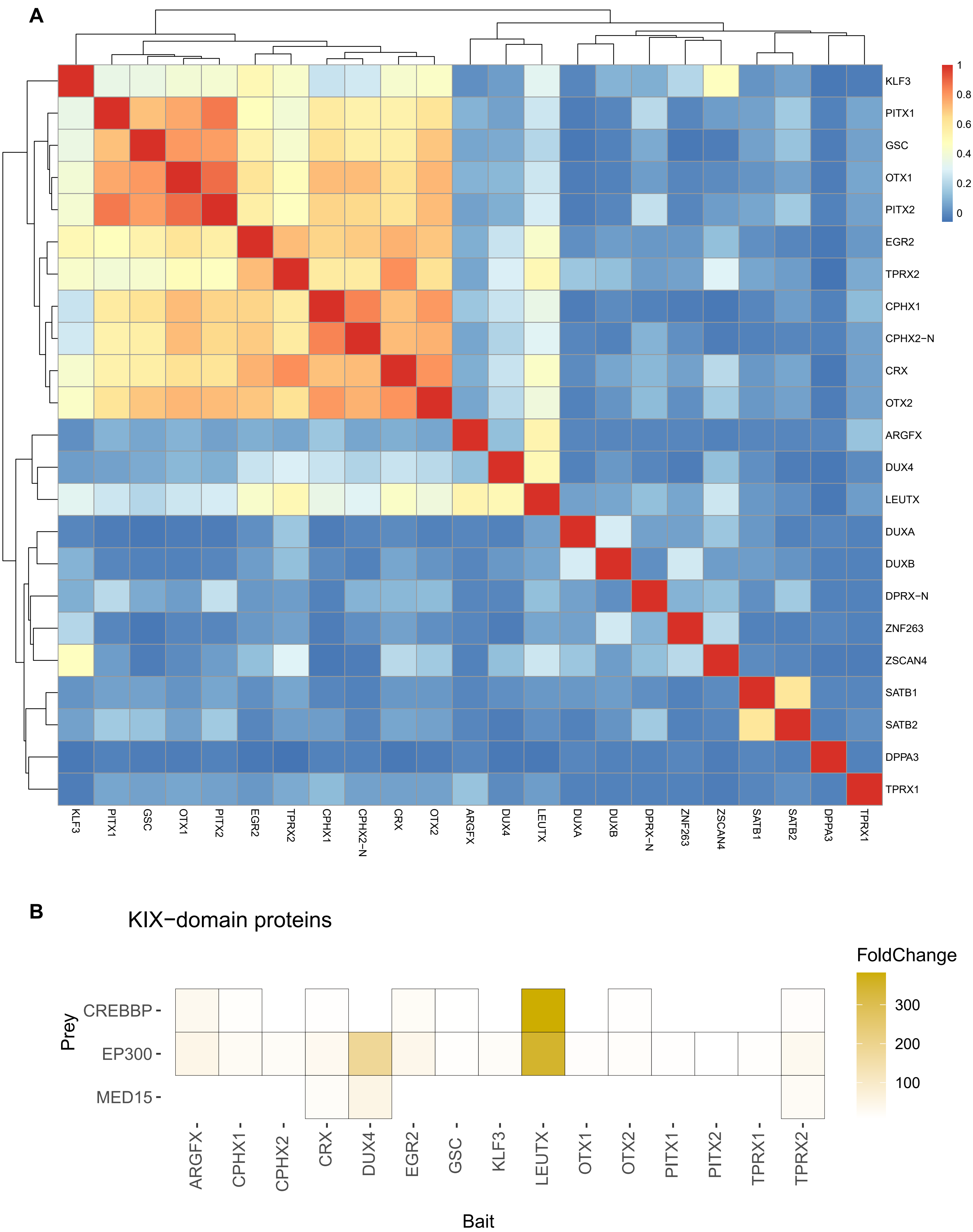

Appendix Figure S6

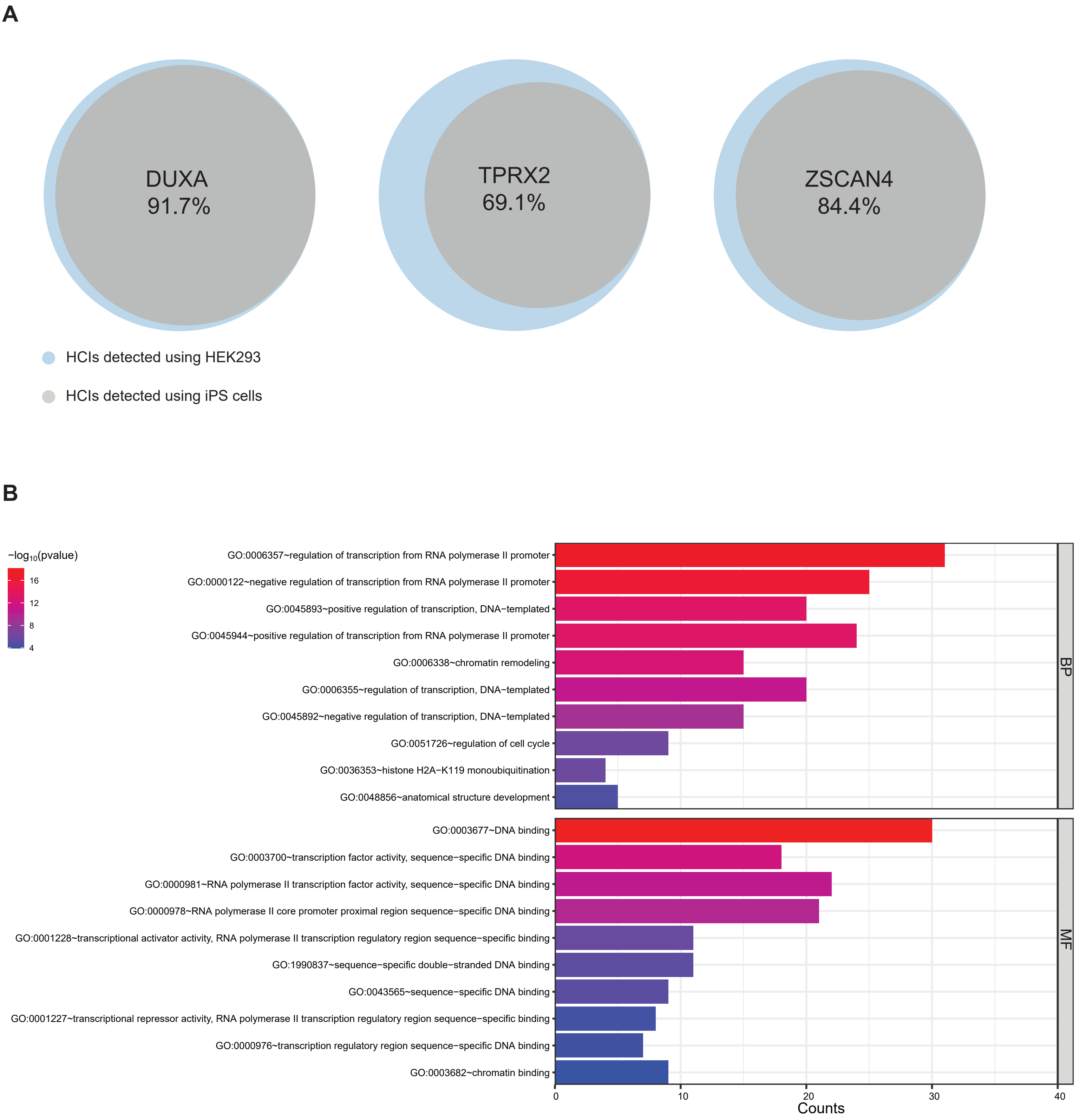

Appendix Figure S7

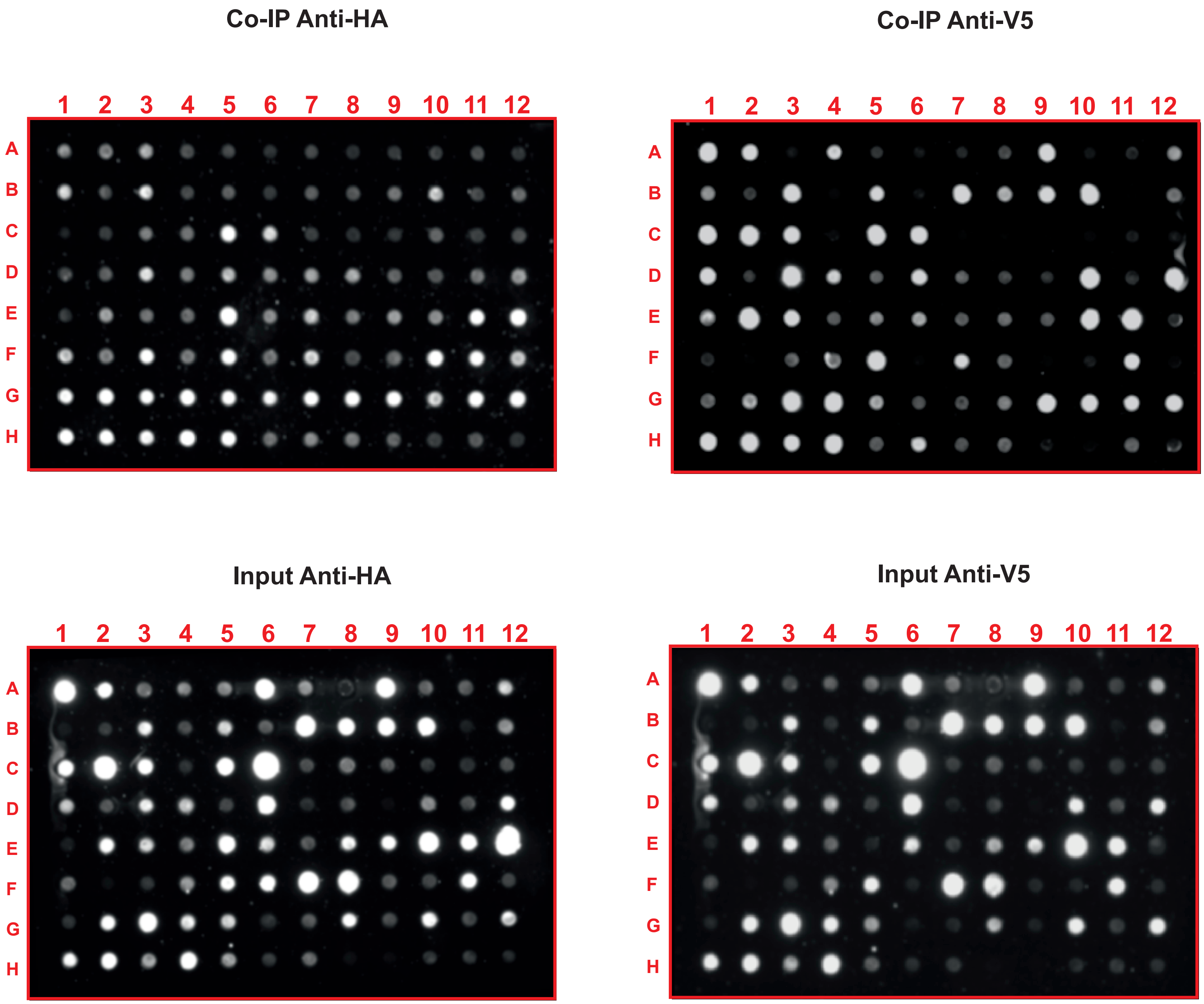

Appendix Figure Legends

Appendix Figure S1. Comparison of difference in control samples with differently tagged GPFs

A) Comparison between NES18 tagged GFP (y-axis) and NLS tagged GFP (x-axis) average spectral counts. **B)** Comparison between MYR tagged GFP (y-axis) and NLS tagged GFP (x-axis) average spectral counts.

Appendix Figure S2. Clustering, localization, and expression analysis of BioID-MS data.

A) tSNE clustering analysis of the total BioID-MS dataset. **B)** MS-microscopy localization based on BioID-MS high-confidence interactions (HCIs) shows localization of baits. Blue gradient depicts localization score. **C)** Gene ontology cellular compartment enrichment of the interactome of each bait. Gradient color depicts enrichment –Log10 FDR. **D)** Expression in ExpressionAtlas. Detection levels of each identified unique prey in ProteinAtlas. The majority of proteins are expressed in most tissues. Only 18 proteins are not typically detected.

Appendix Figure S3. Expression analysis of BioID-MS HCIs

A) Embryonic Stem Cells Atlas of Pluripotency Evidence enrichment analysis of all BioID-MS HCIs, color indicates inverse significant enrichment of pathways (-log10 FDR). Cutoff for image Fishers Exact Test, FDR < 0.05. B) Expression of all BioID-MS HCIs in HEK293 cells. HEK293 expression information obtained from ExpressionAtlas, with cutoff for detection NX>= 1. C) Expression of each gene corresponding to identified BioID-MS HCIs in the embryonic sequencing data sets Zou et al. 2022, Yan et al. 2013, and Petropolous et al. 2016, wherein cutoff for expression is as per in the original paper and the results reflect only those genes expressed in the relevant cell stage specific dataset (8-cell stage for Zou et al. 2022, Yan et al. 2013 and E3 for Petropolous et al. 2016). Further, expression data of same genes in HEK293s from ExpressionAtlas is added. Majority of detected BioID-MS HCIs (208/347, 60%) are detected in all datasets.D) Expression of each gene corresponding to identified BioID-MS HCIs in the embryonic sequencing data sets Zou et al. 2022, Yan et al. 2013, and Petropoulos et al. 2016, wherein cutoff for expression is as per in the original paper and the results reflect only those genes expressed in the relevant cell stage specific dataset (ICMl stage for Zou et al. 2022, Yan et al. 2013 and E4 for Petropolous et al. 2016). Further, expression data of same genes in HEK293s from ExpressionAtlas is added. Majority of detected BioID-MS HCIs (191/347, 55%) are detected in all datasets.

Appendix Figure S4. Further expression analysis of BioID-MS HCIs

A) More detailed expression plots of all detected BioID-MS HCIs conpared to each embryonic transcriptomic dataset including all cell stages for Zou et al. 2022, Yan et al. 2013, and Petropoulos et al. 2016. Further, comparison with 8-cell like cell datasets Yoshihara et al. 2022 iBM-genes (referred to as Yoshihara iBM), Mazid et al. 2022 8CLC hub genes (referred to as Mazid hub), Taubenschmid-Stowers et al. 2022 8CLC marker genes (referred to as TS 8CLC markers) and Yu et al. 2022 8CLC genes (referred to as Yu 8CLC genes). B) Totality of all expressed genes in HEK293 (ExpressionAtlas), Mazid et al. 2022 8CLC hub genes (Mazid hub) and Yoshihara et al. IBM-genes (Yoshihara iBM). C) Venn diagram of the genes detected as BioID-MS HCIs and as Mazid et al. 2022 hub genes, Yoshihara et al. 2022 iBM-genes or expressed in HEK293s (ExpressionAtlas). Majority of BioID-MS HCIs (271) are not 8CLC hub genes or iBM-genes. 39 BioID-MS HCIs are Mazid et al. 2022 hub genes and 19 are Yoshihara et al. 2022 iBM-genes. Out of these, 14 are only detected in Yoshihara et al. 2022, 5 are

shared between Mazid et al. 2022 and Yoshihara et al. 2022, and 3 are only detected in Mazid et al. 2022 and not in HEK293s.

Appendix Figure S5. Expansion of BioID-MS dataset with other embryonic factors LEUTX and DUX4.

A) Bait-Bait correlation analysis (Pearson) with phylogenetic tree based on similarity of Prey interaction AvgSpec (Eucledian distance) of the baits. Red color corresponds to high correlation (Peason coefficient > 0.8) and blue low correlation (Pearson coefficient < 0.2). B) Interaction of the expanded BioID dataset with KIX-domain containing proteins CREBBP, EP300 and MED16. Strenght of mustard color reflects FoldChange of AvgSpec between sample and control.

Appendix Figure S6. Validation of the interactome data with iPSC cells

A) Venn diagrams represent the percentage of detected proteins DUXA, TPRX2, and ZSCAN4 in the HEL24.3 cell line. The overlapping regions between the circles indicate proteins detected using both HEK293 and iPS cells. Individual regions specify the proteins exclusively detected using HEK293 cell lines. B) Gene Ontology Biological Processes (GO-BP) and Molecular Functions (GO-MF) analysis of the TPRX2 interactors specifically detected in the HEK293 cells (Fishers Exact Test, FDR < 0.05).

Appendix Figure S7. Validation of interaction data by Co-IP and dot blotting

Proteomics interaction data were evaluated by Co-IP and dot blotting, related to Supplementary Table S8. In total, 96 selected interaction pairs from analysed using co-expression Co-IP, of which 79 pairs (82%) showed positive signal. The bait proteins tagged with a Strep-HA and prey proteins tagged with V5 were co-expressed in HEK293 cells. Strep-HA-tagged proteins were immunoprecipitated with the Strep-Tactin Sepharose resin, and then, the immunoprecipitated complexes were analyzed by dot blotting with anti-V5 antibody and anti-HA antibody, respectively. Five percent of the total cell lysate was used as the input loading control.
