# Supplementary material for: Interaction network of human early embryonic transcription factors
Source: EMBO Rep. 2024 Jan 31;25(3):1589–622. doi: 10.1038/s44319-024-00074-0 (PMC10933267; doi:10.1038/s44319-024-00074-0)
Supplement: Supplementary file 12 — Expanded View Figures [file 44319_2024_74_MOESM12_ESM.pdf]

Expanded View Figures

Figure EV1. Additional info and methods used in this study.

(**A**) Expression of other baits in the bait set in the embryonic transcriptomics dataset Yan et al (2013). (**B**) Overview of the methods used in this study. We first produced a stable cell line containing the protein of interest (bait), and then performed affinity purification mass spectrometry using two methods and chromatin immunoprecipitation sequencing in the same HEK293 cell line. We combined the produced data and performed bioinformatic analysis. (**C**) Domain prediction for CPHX1 and CPHX2. (**D**) Homeodomain alignment of all homeodomain proteins in the dataset.

 © The Author(s)

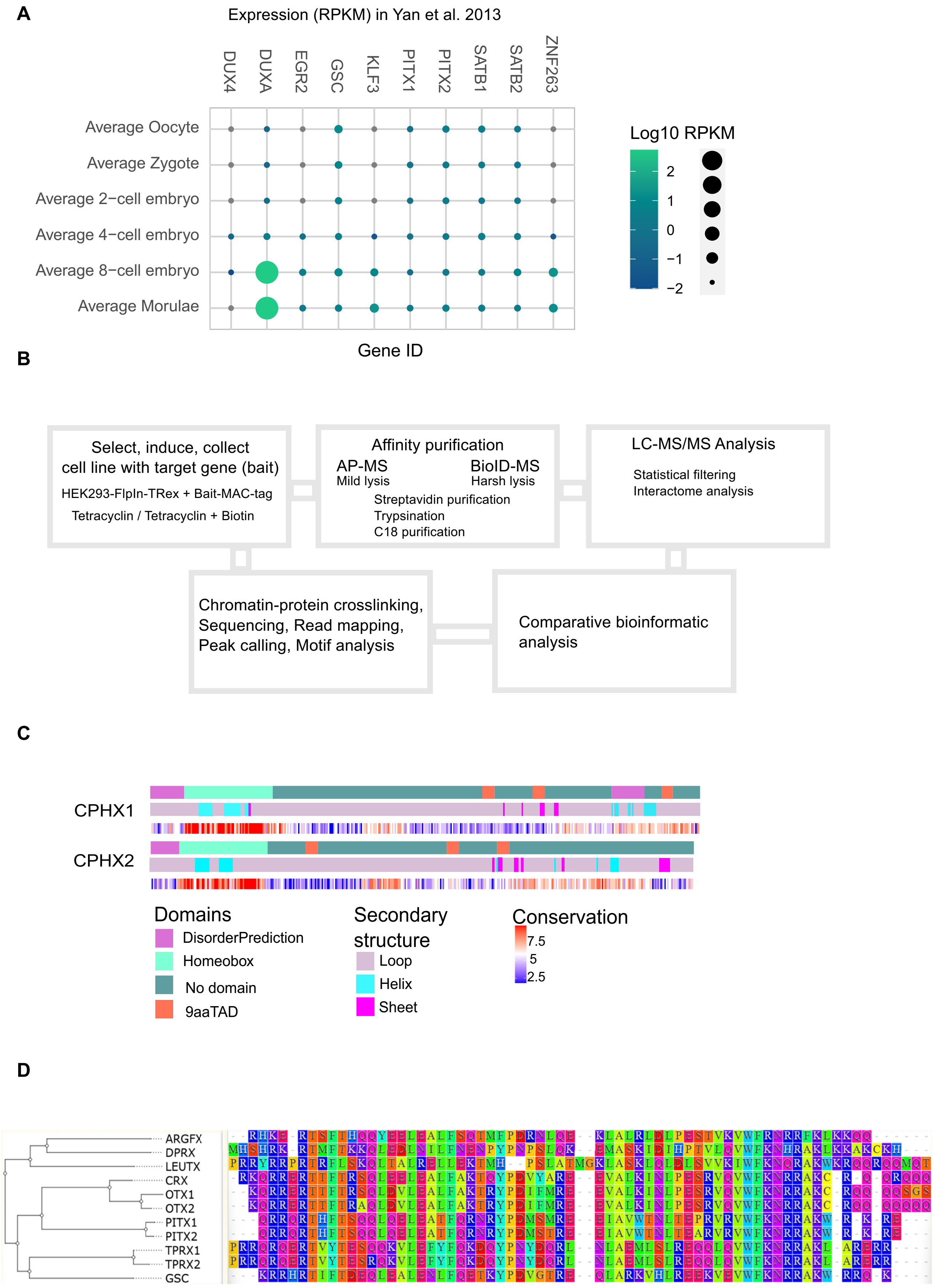
A
Expression (RPKM) in Yan et al. 2013
DUX4
DUXA
EGR2
GSC
KLF3
PITX1
PITX2
SATB1
SATB2
ZNF263
Average Oocyte
Average Zygote
Average 2−cell embryo
Average 4−cell embryo
Average 8−cell embryo
Average Morulae
Gene ID
Log10 RPKM
2
1
0
−1
−2
B
Select, induce, collect cell line with target gene (bait)
HEK293-FlpIn-TRex + Bait-MAC-tag
Tetracyclin / Tetracyclin + Biotin
Affinity purification
AP-MS
Mild lysis
BioID-MS
Harsh lysis
Streptavidin purification
Trypsination
C18 purification
LC-MS/MS Analysis
Statistical filtering
Interactome analysis
Chromatin-protein crosslinking, Sequencing, Read mapping, Peak calling, Motif analysis
Comparative bioinformatic analysis
C
CPHX1
CPHX2
Domains
DisorderPrediction
Homeobox
No domain
9aaTAD
Secondary structure
Loop
Helix
Sheet
Conservation
7.5
5
2.5
D
ARGFX
DPRX
LEUTX
CRX
OTX1
OTX2
PITX1
PITX2
TPRX1
TPRX2
GSC

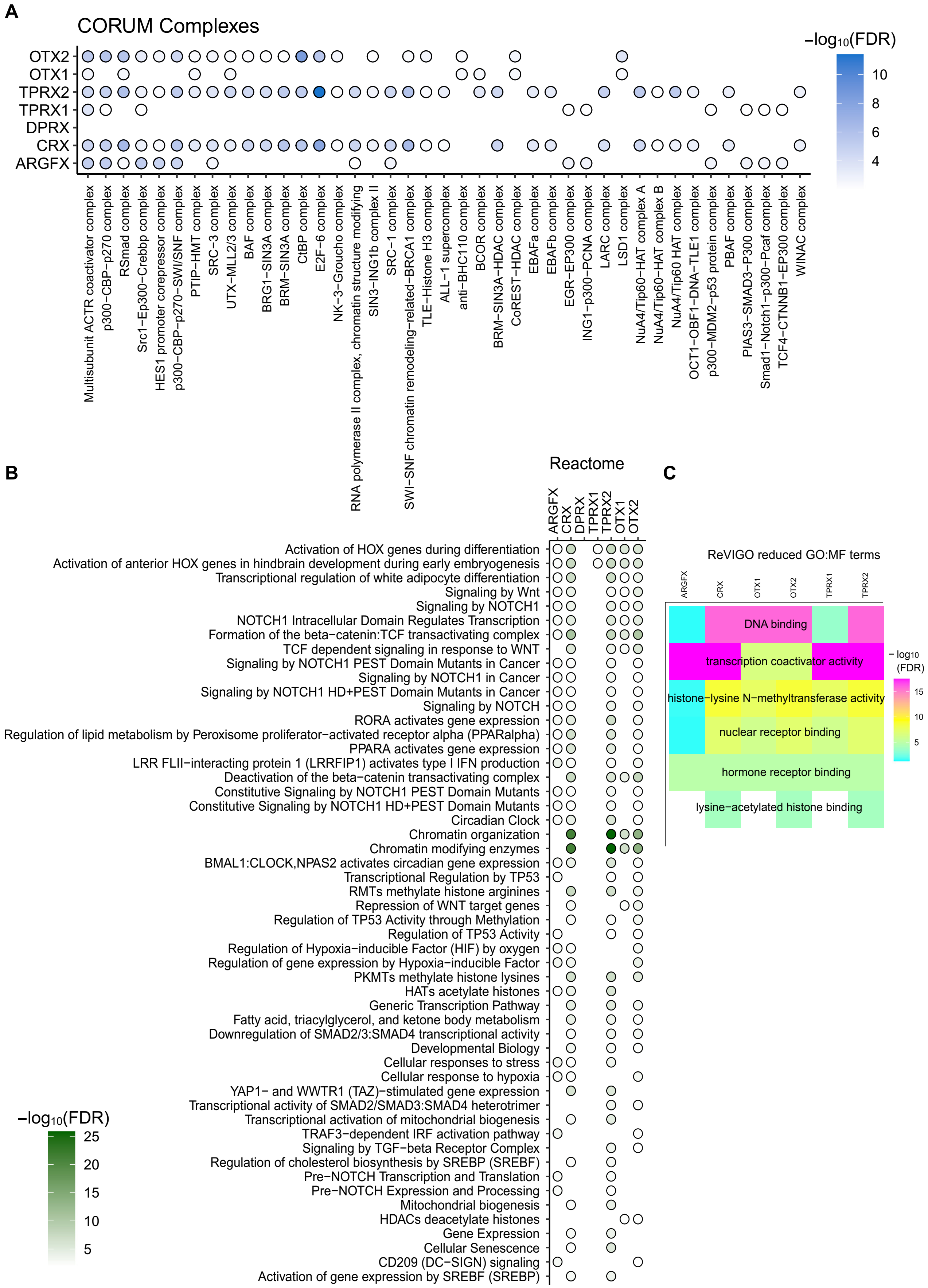
A
CORUM Complexes
−log10(FDR)
10
8
6
4
OTX2
OTX1
TPRX2
TPRX1
DPRX
CRX
ARGFX
Multisubunit ACTR coactivator complex
p300−CBP−p270 complex
RSmad complex
Src1−Ep300−Crebbp complex
HES1 promoter corepressor complex
p300−CBP−p270−SWI/SNF complex
PTIP−HMT complex
SRC−3 complex
UTX−MLL2/3 complex
BAF complex
BRG1−SIN3A complex
BRM−SIN3A complex
CtBP complex
E2F−6 complex
NK−3−Groucho complex
RNA polymerase II complex, chromatin structure modifying
SIN3−ING1b complex II
SRC−1 complex
SWI−SNF chromatin remodeling−related−BRCA1 complex
TLE−Histone H3 complex
ALL−1 supercomplex
anti−BHC110 complex
BCOR complex
BRM−SIN3A−HDAC complex
CoREST−HDAC complex
EBAFa complex
EBAFb complex
EGR−EP300 complex
ING1−p300−PCNA complex
LARC complex
LSD1 complex
NuA4/Tip60−HAT complex A
NuA4/Tip60−HAT complex B
NuA4/Tip60 HAT complex
OCT1−OBF1−DNA−TLE1 complex
p300−MDM2−p53 protein complex
PBAF complex
PIAS3−SMAD3−P300 complex
Smad1−Notch1−p300−Pcaf complex
TCF4−CTNNB1−EP300 complex
WINAC complex
B
Reactome
ARGFX
CRX
DPRX
TPRX1
TPRX2
OTX1
OTX2
Activation of HOX genes during differentiation
Activation of anterior HOX genes in hindbrain development during early embryogenesis
Transcriptional regulation of white adipocyte differentiation
Signaling by Wnt
Signaling by NOTCH1
NOTCH1 Intracellular Domain Regulates Transcription
Formation of the beta−catenin:TCF transactivating complex
TCF dependent signaling in response to WNT
Signaling by NOTCH1 PEST Domain Mutants in Cancer
Signaling by NOTCH1 in Cancer
Signaling by NOTCH1 HD+PEST Domain Mutants in Cancer
Signaling by NOTCH
RORA activates gene expression
Regulation of lipid metabolism by Peroxisome proliferator−activated receptor alpha (PPARalpha)
PPARA activates gene expression
LRR FLII−interacting protein 1 (LRRFIP1) activates type I IFN production
Deactivation of the beta−catenin transactivating complex
Constitutive Signaling by NOTCH1 PEST Domain Mutants
Constitutive Signaling by NOTCH1 HD+PEST Domain Mutants
Circadian Clock
Chromatin organization
Chromatin modifying enzymes
BMAL1:CLOCK,NPAS2 activates circadian gene expression
Transcriptional Regulation by TP53
RMTs methylate histone arginines
Repression of WNT target genes
Regulation of TP53 Activity through Methylation
Regulation of TP53 Activity
Regulation of Hypoxia−inducible Factor (HIF) by oxygen
Regulation of gene expression by Hypoxia−inducible Factor
PKMTs methylate histone lysines
HATs acetylate histones
Generic Transcription Pathway
Fatty acid, triacylglycerol, and ketone body metabolism
Downregulation of SMAD2/3:SMAD4 transcriptional activity
Developmental Biology
Cellular responses to stress
Cellular response to hypoxia
YAP1− and WWTR1 (TAZ)−stimulated gene expression
Transcriptional activity of SMAD2/SMAD3:SMAD4 heterotrimer
Transcriptional activation of mitochondrial biogenesis
TRAF3−dependent IRF activation pathway
Signaling by TGF−beta Receptor Complex
Regulation of cholesterol biosynthesis by SREBP (SREBF)
Pre−NOTCH Transcription and Translation
Pre−NOTCH Expression and Processing
Mitochondrial biogenesis
HDACs deacetylate histones
Gene Expression
Cellular Senescence
CD209 (DC−SIGN) signaling
Activation of gene expression by SREBF (SREBP)
−log10(FDR)
25
20
15
10
5
C
ReViGO reduced GO:MF terms
ARGFX
CRX
OTX1
OTX2
TPRX1
TPRX2
DNA binding
transcription coactivator activity
histone−lysine N−methyltransferase activity
nuclear receptor binding
hormone receptor binding
lysine−acetylated histone binding
− log10 (FDR)
15
10
5

 © The Author(s)

Figure EV2. Enrichment analysis of PRDL bait interactomes.

(**A**) CORUM complex enrichment analysis for PRDL bait interactomes, color indicates inverse significant enrichment of complex ($-\log_{10}$ (FDR)). Cutoff for image FDR < 0.05, Fishers Exact Test. (**B**) Reactome pathway enrichment analysis for PRDL bait interactomes, color indicates inverse significant enrichment of pathways ($-\log_{10}$ (FDR)). Cutoff for image FDR < 0.05´, Fishers Exact Test. (**C**) Gene ontology molecular function enrichment analysis of PRDL bait interactomes, color indicates inverse significant enrichment of pathways ($-\log_{10}$ (FDR)). Cutoff for image FDR < 0.05, Fishers Exact Test.

© The Author(s)

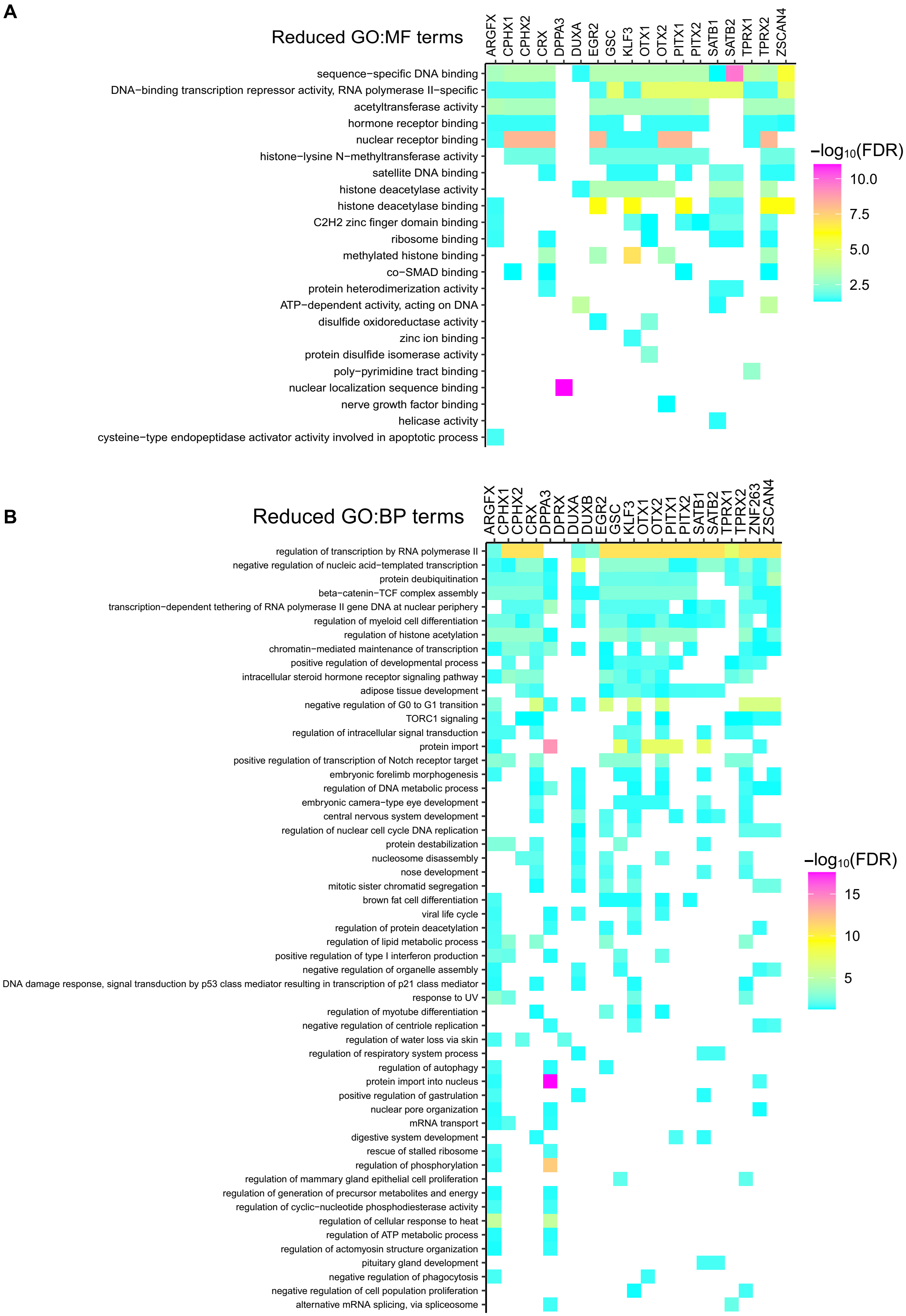
A
Reduced GO:MF terms
ARGFX
CPHX1
CPHX2
CRX
DPPA3
DUXA
EGR2
GSC
KLF3
OTX1
OTX2
PITX1
PITX2
SATB1
SATB2
TPRX1
TPRX2
ZSCAN4
sequence−specific DNA binding
DNA−binding transcription repressor activity, RNA polymerase II−specific
acetyltransferase activity
hormone receptor binding
nuclear receptor binding
histone−lysine N−methyltransferase activity
satellite DNA binding
histone deacetylase activity
histone deacetylase binding
C2H2 zinc finger domain binding
ribosome binding
methylated histone binding
co−SMAD binding
protein heterodimerization activity
ATP−dependent activity, acting on DNA
disulfide oxidoreductase activity
zinc ion binding
protein disulfide isomerase activity
poly−pyrimidine tract binding
nuclear localization sequence binding
nerve growth factor binding
helicase activity
cysteine−type endopeptidase activator activity involved in apoptotic process
−log10(FDR)
10.0
7.5
5.0
2.5
B
Reduced GO:BP terms
ARGFX
CPHX1
CPHX2
CRX
DPPA3
DPRX
DUXA
DUXB
EGR2
GSC
KLF3
OTX1
OTX2
PITX1
PITX2
SATB1
SATB2
TPRX1
TPRX2
ZNF263
ZSCAN4
regulation of transcription by RNA polymerase II
negative regulation of nucleic acid−templated transcription
protein deubiquitination
beta−catenin−TCF complex assembly
transcription−dependent tethering of RNA polymerase II gene DNA at nuclear periphery
regulation of myeloid cell differentiation
regulation of histone acetylation
chromatin−mediated maintenance of transcription
positive regulation of developmental process
intracellular steroid hormone receptor signaling pathway
adipose tissue development
negative regulation of G0 to G1 transition
TORC1 signaling
regulation of intracellular signal transduction
protein import
positive regulation of transcription of Notch receptor target
embryonic forelimb morphogenesis
regulation of DNA metabolic process
embryonic camera−type eye development
central nervous system development
regulation of nuclear cell cycle DNA replication
protein destabilization
nucleosome disassembly
nose development
mitotic sister chromatid segregation
brown fat cell differentiation
viral life cycle
regulation of protein deacetylation
regulation of lipid metabolic process
positive regulation of type I interferon production
negative regulation of organelle assembly
DNA damage response, signal transduction by p53 class mediator resulting in transcription of p21 class mediator
response to UV
regulation of myotube differentiation
negative regulation of centriole replication
regulation of water loss via skin
regulation of respiratory system process
regulation of autophagy
protein import into nucleus
positive regulation of gastrulation
nuclear pore organization
mRNA transport
digestive system development
rescue of stalled ribosome
regulation of phosphorylation
regulation of mammary gland epithelial cell proliferation
regulation of generation of precursor metabolites and energy
regulation of cyclic−nucleotide phosphodiesterase activity
regulation of cellular response to heat
regulation of ATP metabolic process
regulation of actomyosin structure organization
pituitary gland development
negative regulation of phagocytosis
negative regulation of cell population proliferation
alternative mRNA splicing, via spliceosome
−log10(FDR)
15
10
5

 © The Author(s)

Figure EV3. Gene ontology enrichment analysis of BioID-MS HCIs.

(**A**) Gene ontology molecular function enrichment analysis of all BioID-MS HCIs, color indicates inverse significant enrichment of pathways ($-\log_{10}$ (FDR)). Cutoff for image FDR < 0.05, Fishers Exact Test. (**B**) Gene ontology biological process enrichment analysis of all BioID-MS HCIs, color indicates inverse significant enrichment of pathways ($-\log_{10}$ (FDR)). Cutoff for image FDR < 0.05, Fishers Exact Test.

© The Author(s)

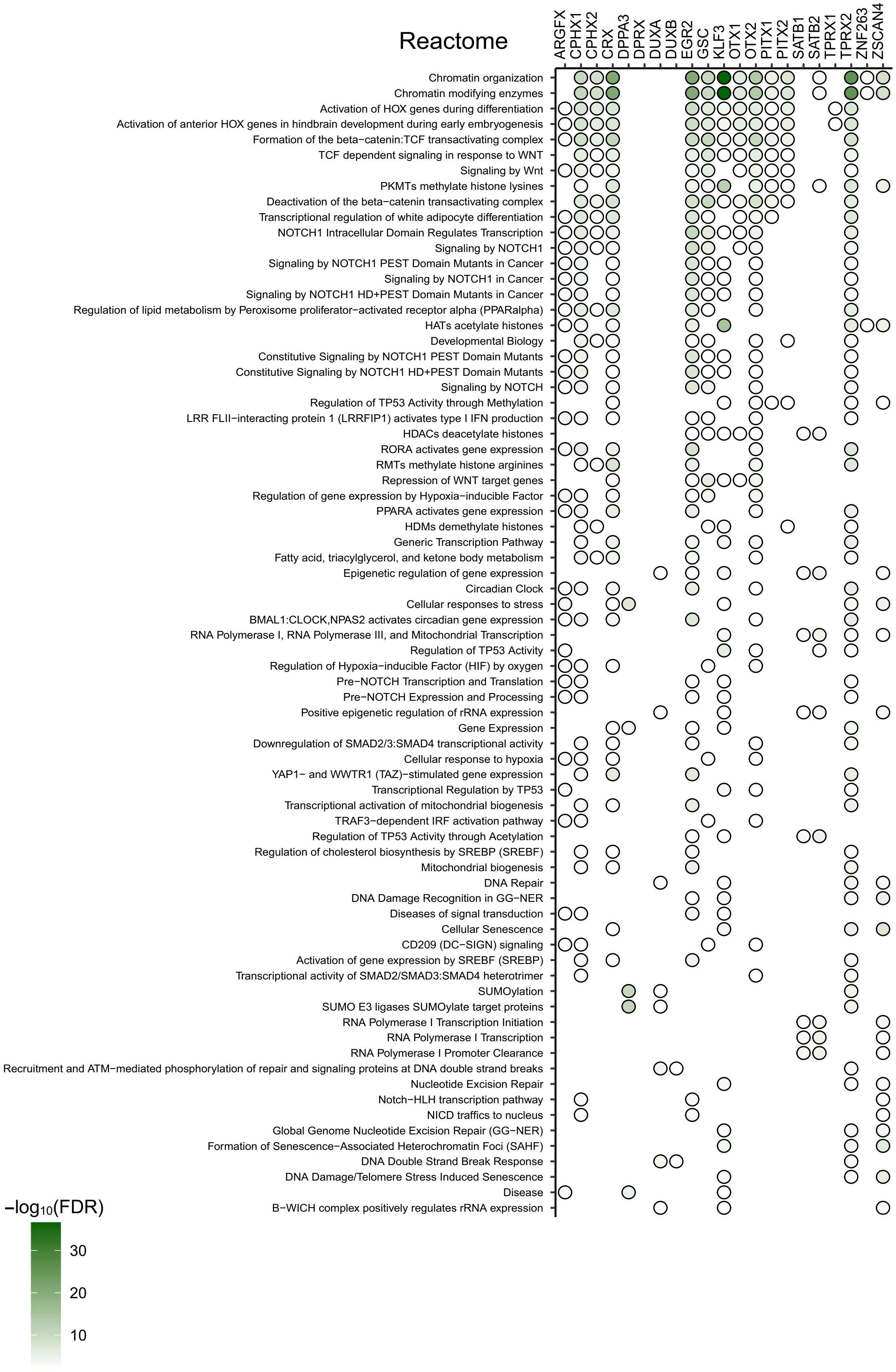
Reactome
ARGFX
CPHX1
CPHX2
CRX
DPPA3
DPRX
DUXA
DUXB
EGR2
GSC
KLF3
OTX1
OTX2
PITX1
PITX2
SATB1
SATB2
TPRX1
TPRX2
ZNF263
ZSCAN4
Chromatin organization
Chromatin modifying enzymes
Activation of HOX genes during differentiation
Activation of anterior HOX genes in hindbrain development during early embryogenesis
Formation of the beta−catenin:TCF transactivating complex
TCF dependent signaling in response to WNT
Signaling by Wnt
PKMTs methylate histone lysines
Deactivation of the beta−catenin transactivating complex
Transcriptional regulation of white adipocyte differentiation
NOTCH1 Intracellular Domain Regulates Transcription
Signaling by NOTCH1
Signaling by NOTCH1 PEST Domain Mutants in Cancer
Signaling by NOTCH1 in Cancer
Signaling by NOTCH1 HD+PEST Domain Mutants in Cancer
Regulation of lipid metabolism by Peroxisome proliferator−activated receptor alpha (PPARalpha)
HATs acetylate histones
Developmental Biology
Constitutive Signaling by NOTCH1 PEST Domain Mutants
Constitutive Signaling by NOTCH1 HD+PEST Domain Mutants
Signaling by NOTCH
Regulation of TP53 Activity through Methylation
LRR FLII−interacting protein 1 (LRRFIP1) activates type I IFN production
HDACs deacetylate histones
RORA activates gene expression
RMTs methylate histone arginines
Repression of WNT target genes
Regulation of gene expression by Hypoxia−inducible Factor
PPARA activates gene expression
HDMs demethylate histones
Generic Transcription Pathway
Fatty acid, triacylglycerol, and ketone body metabolism
Epigenetic regulation of gene expression
Circadian Clock
Cellular responses to stress
BMAL1:CLOCK,NPAS2 activates circadian gene expression
RNA Polymerase I, RNA Polymerase III, and Mitochondrial Transcription
Regulation of TP53 Activity
Regulation of Hypoxia−inducible Factor (HIF) by oxygen
Pre−NOTCH Transcription and Translation
Pre−NOTCH Expression and Processing
Positive epigenetic regulation of rRNA expression
Gene Expression
Downregulation of SMAD2/3:SMAD4 transcriptional activity
Cellular response to hypoxia
YAP1− and WWTR1 (TAZ)−stimulated gene expression
Transcriptional Regulation by TP53
Transcriptional activation of mitochondrial biogenesis
TRAF3−dependent IRF activation pathway
Regulation of TP53 Activity through Acetylation
Regulation of cholesterol biosynthesis by SREBP (SREBF)
Mitochondrial biogenesis
DNA Repair
DNA Damage Recognition in GG−NER
Diseases of signal transduction
Cellular Senescence
CD209 (DC−SIGN) signaling
Activation of gene expression by SREBF (SREBP)
Transcriptional activity of SMAD2/SMAD3:SMAD4 heterotrimer
SUMOylation
SUMO E3 ligases SUMOylate target proteins
RNA Polymerase I Transcription Initiation
RNA Polymerase I Transcription
RNA Polymerase I Promoter Clearance
Recruitment and ATM−mediated phosphorylation of repair and signaling proteins at DNA double strand breaks
Nucleotide Excision Repair
Notch−HLH transcription pathway
NICD traffics to nucleus
Global Genome Nucleotide Excision Repair (GG−NER)
Formation of Senescence−Associated Heterochromatin Foci (SAHF)
DNA Double Strand Break Response
DNA Damage/Telomere Stress Induced Senescence
Disease
B−WICH complex positively regulates rRNA expression
−log10(FDR)
30
20
10

 © The Author(s)

Figure EV4. Reactome pathway and ChIP-seq peak overlap analysis.

Reactome pathway enrichment analysis of all bait interactomes, color indicates inverse significant enrichment of pathways ($-\log_{10}$ (FDR)). Cutoff for image FDR < 0.05, Fishers Exact Test.

© The Author(s)

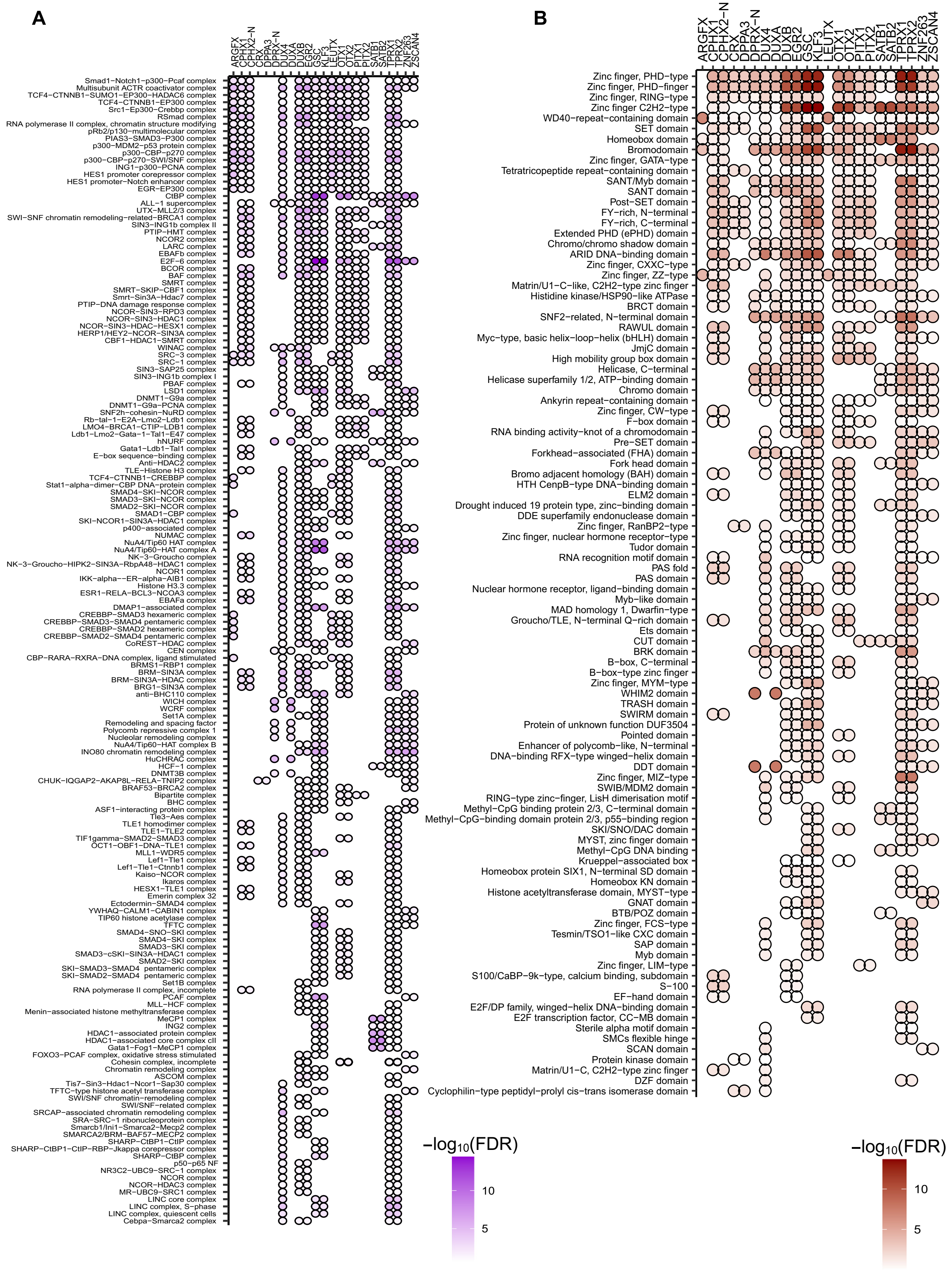
A
ARGFX
CPHX1
CPHX2−N
CRX
DPPA3
DPRX−N
DUX4
DUXA
DUXB
EGR2
GSC
KLF3
LEUTX
OTX1
OTX2
PITX1
PITX2
SATB1
SATB2
TPRX1
TPRX2
ZNF263
ZSCAN4
Smad1−Notch1−p300−Pcaf complex
Multisubunit ACTR coactivator complex
TCF4−CTNNB1−SUMO1−EP300−HADAC6 complex
TCF4−CTNNB1−EP300 complex
Src1−Ep300−Crebbp complex
RSmad complex
RNA polymerase II complex, chromatin structure modifying
pRb2/p130−multimolecular complex
PIAS3−SMAD3−P300 complex
p300−MDM2−p53 protein complex
p300−CBP−p270 complex
p300−CBP−p270−SWI/SNF complex
ING1−p300−PCNA complex
HES1 promoter corepressor complex
HES1 promoter−Notch enhancer complex
EGR−EP300 complex
CtBP complex
ALL−1 supercomplex
UTX−MLL2/3 complex
SWI−SNF chromatin remodeling−related−BRCA1 complex
SIN3−ING1b complex II
PTIP−HMT complex
NCOR2 complex
LARC complex
EBAFb complex
E2F−6 complex
BCOR complex
BAF complex
SMRT complex
SMRT−SKIP−CBF1 complex
Smrt−Sin3A−Hdac7 complex
PTIP−DNA damage response complex
NCOR−SIN3−RPD3 complex
NCOR−SIN3−HDAC1 complex
NCOR−SIN3−HDAC−HESX1 complex
HERP1/HEY2−NCOR−SIN3A complex
CBF1−HDAC1−SMRT complex
WINAC complex
SRC−3 complex
SRC−1 complex
SIN3−SAP25 complex
SIN3−ING1b complex I
PBAF complex
LSD1 complex
DNMT1−G9a complex
DNMT1−G9a−PCNA complex
SNF2h−cohesin−NuRD complex
Rb−tal−1−E2A−Lmo2−Ldb1 complex
LMO4−BRCA1−CTIP−LDB1 complex
Ldb1−Lmo2−Gata−1−Tal1−E47 complex
hNURF complex
Gata1−Ldb1−Tal1 complex
E−box sequence−binding complex
Anti−HDAC2 complex
TLE−Histone H3 complex
TCF4−CTNNB1−CREBBP complex
Stat1−alpha−dimer−CBP DNA−protein complex
SMAD4−SKI−NCOR complex
SMAD3−SKI−NCOR complex
SMAD2−SKI−NCOR complex
SMAD1−CBP complex
SKI−NCOR1−SIN3A−HDAC1 complex
p400−associated complex
NUMAC complex
NuA4/Tip60 HAT complex
NuA4/Tip60−HAT complex A
NK−3−Groucho complex
NK−3−Groucho−HIPK2−SIN3A−RbpA48−HDAC1 complex
NCOR1 complex
IKK−alpha−−ER−alpha−AIB1 complex
Histone H3.3 complex
ESR1−RELA−BCL3−NCOA3 complex
EBAFa complex
DMAP1−associated complex
CREBBP−SMAD3 hexameric complex
CREBBP−SMAD3−SMAD4 pentameric complex
CREBBP−SMAD2 hexameric complex
CREBBP−SMAD2−SMAD4 pentameric complex
CoREST−HDAC complex
CEN complex
CBP−RARA−RXRA−DNA complex, ligand stimulated
BRMS1−RBP1 complex
BRM−SIN3A complex
BRM−SIN3A−HDAC complex
BRG1−SIN3A complex
anti−BHC110 complex
WICH complex
WCRF complex
Set1A complex
Remodeling and spacing factor
Polycomb repressive complex 1
Nucleolar remodeling complex
NuA4/Tip60−HAT complex B
INO80 chromatin remodeling complex
HuCHRAC complex
HCF−1 complex
DNMT3B complex
CHUK−IQGAP2−AKAP8L−RELA−TNIP2 complex
BRAF53−BRCA2 complex
Bipartite complex
BHC complex
ASF1−interacting protein complex
Tle3−Aes complex
TLE1 homodimer complex
TLE1−TLE2 complex
TIF1gamma−SMAD2−SMAD3 complex
OCT1−OBF1−DNA−TLE1 complex
MLL1−WDR5 complex
Lef1−Tle1 complex
Lef1−Tle1−Ctnnb1 complex
Kaiso−NCOR complex
Ikaros complex
HESX1−TLE1 complex
Emerin complex 32
Ectodermin−SMAD4 complex
YWHAQ−CALM1−CABIN1 complex
TIP60 histone acetylase complex
TFTC complex
SMAD4−SNO−SKI complex
SMAD4−SKI complex
SMAD3−SKI complex
SMAD3−cSKI−SIN3A−HDAC1 complex
SMAD2−SKI complex
SKI−SMAD3−SMAD4 pentameric complex
SKI−SMAD2−SMAD4 pentameric complex
Set1B complex
RNA polymerase II complex, incomplete
PCAF complex
MLL−HCF complex
Menin−associated histone methyltransferase complex
MeCP1 complex
ING2 complex
HDAC1−associated protein complex
HDAC1−associated core complex cII
Gata1−Fog1−MeCP1 complex
FOXO3−PCAF complex, oxidative stress stimulated
Cohesin complex, incomplete
Chromatin remodeling complex
ASCOM complex
Tis7−Sin3−Hdac1−Ncor1−Sap30 complex
TFTC−type histone acetyl transferase complex
SWI/SNF chromatin−remodeling complex
SWI/SNF−related complex
SRCAP−associated chromatin remodeling complex
SRA−SRC−1 ribonucleoprotein complex
Smarcb1/Ini1−Smarca2−Mecp2 complex
SMARCA2/BRM−BAF57−MECP2 complex
SHARP−CtBP1−CtIP complex
SHARP−CtBP1−CtIP−RBP−Jkappa corepressor complex
SHARP−CtBP complex
p50−p65 NF
NR3C2−UBC9−SRC−1 complex
NCOR complex
NCOR−HDAC3 complex
MR−UBC9−SRC1 complex
LINC core complex
LINC complex, S−phase
LINC complex, quiescent cells
Cebpa−Smarca2 complex
−log10(FDR)
10
5
B
ARGFX
CPHX1
CPHX2−N
CRX
DPPA3
DPRX−N
DUX4
DUXA
DUXB
EGR2
GSC
KLF3
LEUTX
OTX1
OTX2
PITX1
PITX2
SATB1
SATB2
TPRX1
TPRX2
ZNF263
ZSCAN4
Zinc finger, PHD−type
Zinc finger, PHD−finger
Zinc finger, RING−type
Zinc finger C2H2−type
WD40−repeat−containing domain
SET domain
Homeobox domain
Bromodomain
Zinc finger, GATA−type
Tetratricopeptide repeat−containing domain
SANT/Myb domain
SANT domain
Post−SET domain
FY−rich, N−terminal
FY−rich, C−terminal
Extended PHD (ePHD) domain
Chromo/chromo shadow domain
ARID DNA−binding domain
Zinc finger, CXXC−type
Zinc finger, ZZ−type
Matrin/U1−C−like, C2H2−type zinc finger
Histidine kinase/HSP90−like ATPase
BRCT domain
SNF2−related, N−terminal domain
RAWUL domain
Myc−type, basic helix−loop−helix (bHLH) domain
JmjC domain
High mobility group box domain
Helicase, C−terminal
Helicase superfamily 1/2, ATP−binding domain
Chromo domain
Ankyrin repeat−containing domain
Zinc finger, CW−type
F−box domain
RNA binding activity−knot of a chromodomain
Pre−SET domain
Forkhead−associated (FHA) domain
Fork head domain
Bromo adjacent homology (BAH) domain
HTH CenpB−type DNA−binding domain
ELM2 domain
Drought induced 19 protein type, zinc−binding domain
DDE superfamily endonuclease domain
Zinc finger, RanBP2−type
Zinc finger, nuclear hormone receptor−type
Tudor domain
RNA recognition motif domain
PAS fold
PAS domain
Nuclear hormone receptor, ligand−binding domain
Myb−like domain
MAD homology 1, Dwarfin−type
Groucho/TLE, N−terminal Q−rich domain
Ets domain
CUT domain
BRK domain
B−box, C−terminal
B−box−type zinc finger
Zinc finger, MYM−type
WHIM2 domain
TRASH domain
SWIRM domain
Protein of unknown function DUF3504
Pointed domain
Enhancer of polycomb−like, N−terminal
DNA−binding RFX−type winged−helix domain
DDT domain
Zinc finger, MIZ−type
SWIB/MDM2 domain
RING−type zinc−finger, LisH dimerisation motif
Methyl−CpG binding protein 2/3, C−terminal domain
Methyl−CpG−binding domain protein 2/3, p55−binding region
SKI/SNO/DAC domain
MYST, zinc finger domain
Methyl−CpG DNA binding
Krueppel−associated box
Homeobox protein SIX1, N−terminal SD domain
Homeobox KN domain
Histone acetyltransferase domain, MYST−type
GNAT domain
BTB/POZ domain
Zinc finger, FCS−type
Tesmin/TSO1−like CXC domain
SAP domain
Myb domain
Zinc finger, LIM−type
S100/CaBP−9k−type, calcium binding, subdomain
S−100
EF−hand domain
E2F/DP family, winged−helix DNA−binding domain
E2F transcription factor, CC−MB domain
Sterile alpha motif domain
SMCs flexible hinge
SCAN domain
Protein kinase domain
Matrin/U1−C, C2H2−type zinc finger
DZF domain
Cyclophilin−type peptidyl−prolyl cis−trans isomerase domain
−log10(FDR)
10
5

◀ **Figure EV5. Enrichment analysis of expanded BioID-MS dataset.**

(**A**) Significantly enriched (Fishers Exact Test, FDR < 0.01) protein complexes in the interactomes of each bait. Protein complexes were obtained from the CORUM database. Only complexes enriched in more than two baits are drawn. Increased blue color indicates inverse statistical significance ($-\log_{10}$ (FDR)). Complexes are ordered by frequency. (**B**) Statistically significant enriched InterPro domains (FDR < 0.01) in the interactome of each bait, indicated by the number of shared baits. Color indicates $-\log_{10}$ (FDR) of enrichment analysis. Domains are ordered by $-\log_{10}$ (FDR) and frequency.

© The Author(s)

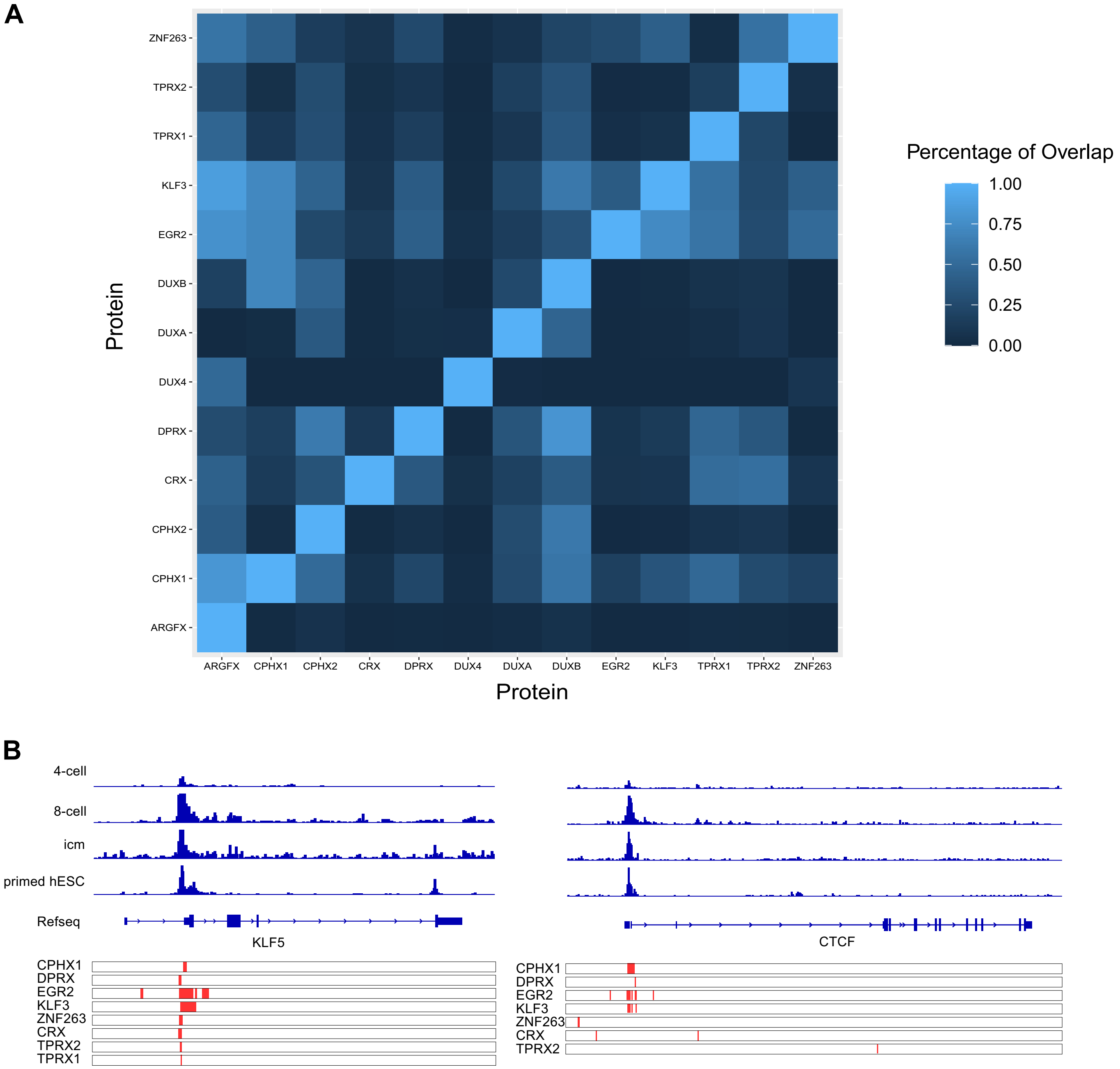

Figure EV6. ChIP-Seq Peaks over key EGA genes.

(**A**) Percentage overlap of identified ChIP-seq peaks. Overlaps were identified using bedtools closest and are shown as percentages from 0 to 1. (**B**) The embryonic ATAC-seq data from Wu et al (2018) from 4-cell, 8-cell, and ICM embryos and primed hESCs over key gene regions identified through our datasets. Genomic locations of differential ChIP-seq peaks are shown in red, relative to their position to key genes (RefSeq annotation).

 © The Author(s)
